# Surveillance of Canine Rabies in the Central African Republic: Impact on Human Health and Molecular Epidemiology

**DOI:** 10.1371/journal.pntd.0004433

**Published:** 2016-02-09

**Authors:** Vianney Tricou, Julie Bouscaillou, Emmanuel Kamba Mebourou, Fidèle Dieudonné Koyanongo, Emmanuel Nakouné, Mirdad Kazanji

**Affiliations:** 1 Laboratoire de Virologie, Institut Pasteur de Bangui, Bangui, Central African Republic; 2 Médecins du Monde France, Paris, France; 3 Service de Santé Publique Vétérinaire, Agence Nationale du Développement de l'Elevage, Bangui, Central African Republic; 4 Laboratoire National de Référence pour la Rage, Institut Pasteur de Bangui, Bangui, Central African Republic; Swiss Tropical and Public Health Institute, SWITZERLAND

## Abstract

**Background:**

Although rabies represents an important public health threat, it is still a neglected disease in Asia and Africa where it causes tens of thousands of deaths annually despite available human and animal vaccines. In the Central African Republic (CAR), an endemic country for rabies, this disease remains poorly investigated.

**Methods:**

To evaluate the extent of the threat that rabies poses in the CAR, we analyzed data for 2012 from the National Reference Laboratory for Rabies, where laboratory confirmation was performed by immunofluorescence and PCR for both animal and human suspected cases, and data from the only anti-rabies dispensary of the country and only place where post-exposure prophylaxis (PEP) is available. Both are located in Bangui, the capital of the CAR. For positive samples, a portion of the N gene was amplified and sequenced to determine the molecular epidemiology of circulating strains.

**Results:**

In 2012, 966 exposed persons visited the anti-rabies dispensary and 632 received a post-exposure rabies vaccination. More than 90% of the exposed persons were from Bangui and its suburbs and almost 60% of them were under 15-years of age. No rabies-related human death was confirmed. Of the 82 samples from suspected rabid dogs tested, 69 were confirmed positive. Most of the rabid dogs were owned although unvaccinated. There was a strong spatiotemporal correlation within Bangui and within the country between reported human exposures and detection of rabid dogs (P<0.001). Phylogenetic analysis indicated that three variants belonging to Africa I and II lineages actively circulated in 2012.

**Conclusions:**

These data indicate that canine rabies was endemic in the CAR in 2012 and had a detrimental impact on human health as shown by the hundreds of exposed persons who received PEP. Implementation of effective public health interventions including mass dog vaccination and improvement of the surveillance and the access to PEP are urgently needed in this country.

## Author Summary

Rabies is a widespread fatal, but preventable, viral disease transmitted from animals to humans. It has been estimated that tens of thousands of people die of rabies annually, mainly in developing countries where rabies is still a neglected disease. In the Central African Republic (CAR), a poor country located at the heart of Africa, rabies is endemic, but its burden remains poorly investigated. Here, we reported a comprehensive analysis of data for 2012 from the Institut Pasteur in Bangui, the capital of the CAR. In 2012, 966 persons reported exposure to suspicious animals, mainly dogs, and 632 received post-exposure rabies vaccination. Most of these people were from Bangui area and were under 15-years of age. Meanwhile, 82 samples from suspected rabid dogs were tested and 69 were confirmed positive. Most of the rabid dogs were owned although unvaccinated. Positive samples were sequenced and we found that three different variants actively circulated in 2012. Theses variants clustered with other viruses found in surroundings countries. Our data suggested that canine rabies was endemic in the CAR with a detrimental impact on human health. We conclude that mass vaccination of domestic dogs and the improvement of the surveillance and the access to post-exposure vaccination are urgently needed to control rabies in the CAR.

## Introduction

Although this fatal disease is preventable since 1884 when Louis Pasteur developed the first vaccine strategy, rabies is still a neglected zoonosis in developing countries where it poses a significant threat to human public health [1]. More than 55,000 people die of rabies every year mostly in Asia and Africa [2]. Rabies virus (RABV) belongs to the genus *Lyssavirus* and family *Rhabdoviridae*. Although all species of mammals are susceptible to rabies virus infection, only a few species are important as reservoirs of infection [3]. The most common route of rabies transmission to humans is the bite of rabid domestic dogs [4]. The WHO considers that canine rabies potentially threatens over three billion people in Asia and Africa [5]. In humans, early clinical features of rabies are nonspecific prodromal symptoms and local neurological symptoms. After an incubation period of variable duration, RABV infects the central nervous system and the clinical presentation of rabies evolves into either encephalitic (furious) or paralytic (dumb) forms [6]. Rabies is almost always fatal once symptoms appear. However, rabies is a 100% vaccine-preventable disease and vaccination can be used in two situations: to protect those who are at risk of exposure to rabies, i.e. pre-exposure prophylaxis (PrEP), and to prevent the development of clinical rabies after exposure has occurred, i.e. post exposure prophylaxis (PEP). Moreover, mass vaccination of domesticated and wild animals is also possible [7]. With a basic reproductive ratio of less than two, canine rabies is an ideal candidate for worldwide elimination [8]. Consequently, canine rabies elimination is the key towards ultimate reduction of the disease burden in humans, as illustrated in Europe and North America, and mass vaccination of dogs is the most cost-effective way to achieve it [1, 5].

In many African countries, dog vaccination programs to date have been inadequate and failed to reduce the incidence of canine rabies [9]. However, it has been shown that effective vaccination campaigns reaching a sufficient percentage of the canine population to potentially eliminate disease and prevent future outbreaks are feasible at a cost that is economically and logistically sustainable in developing countries [9, 10]. For instance, in a study conducted in Tanzania, vaccination of 60–70% of dogs has been sufficient to control dog rabies in the studied area and to significantly reduce demand for human post-exposure rabies treatment [11]. In another study using a model parameterized with routine data on rabid dog and exposed human cases from N’Djaména in Chad, Zinsstag et al. have predicted that a single parenteral rabies mass vaccination of 70% of the dog population would be the most beneficial and cost-effective intervention and would be sufficient to interrupt transmission of rabies to humans for at least six years [12]. This study has also predicted that dog vaccination campaigns combined with human PEP would be more cost-effective compared to human PEP alone beyond a time frame of seven years.

In Africa, previous molecular epidemiological studies have shown that at least four clades are circulating [13–15]. In Central Africa, RABV strains belong to the Africa I and Africa II clades [13, 16]. The Africa I clade is very similar to current Eurasian RABV lineages and is usually grouped into a larger Cosmopolitan clade [14, 17]. The Africa II clade includes RABV strains that circulate in dogs in several Central and Western African countries [16].

The Central African Republic (CAR) is a landlocked country in Central Africa. Its capital is Bangui. It is one of the poorest countries in the world and it has been affected by political instability and internal conflicts for several decades. In the CAR, rabies has been a notifiable disease since April 2009. The surveillance mainly consists of observation of suspicious animals and brain sample collection by the national veterinary services, and laboratory confirmation by the National Reference Laboratory for Rabies. The national veterinary services operate under the National Agency for Livestock Development (ANDE) through an extended network of veterinarians and livestock technicians distributed in 122 sentinel sites across the country. The National Reference Laboratory for Rabies is located at the Institut Pasteur in Bangui. Between August 2006 and December 2008, 86 animal samples (82 of dog origin) of 101 tested positive for rabies [18]. During the same period, seven human cases were recorded and, biologically confirmation was obtained for three of them [18]. Molecular epidemiological studies have shown the co-circulation in the CAR of strains that belong to Africa I and Africa II clades [13, 16]. Oscillations in the numbers of rabid dogs have been observed in Bangui with periods of absence or low circulation and then some increases every five years (manuscript in preparation). This pattern might be comparable to the oscillations observed by Hampson et al. in Southern and Eastern Africa [19].

Public strategies for preventing rabies have been very limited in the CAR. Animal vaccination is only at the dog owner's initiative but it is expensive for most people and rarely done. The only serious attempt to control the disease was episodic mass euthanasia of stray dogs in Bangui. In the whole CAR, there is only one anti-rabies dispensary. This dispensary is located at the Institut Pasteur in Bangui and the available post-exposure rabies prophylaxis consists only in vaccination after exposure and is freely available. Administration of immunoglobulin is exceptionally available through some non-governmental organizations (NGOs) for some very severe exposures. The persons exposed to suspicious rabid animals are usually referred to the anti-rabies dispensary by the veterinary services, as the first place visited by these persons is often a veterinary clinic. The assessment whether the post-exposure vaccination is needed is usually made by a livestock technician or veterinarian based on the type and circumstances of the exposure. When possible, the veterinary services put the animal under observation and depending on the evolution of the animal health status, they can eventually advise the anti-rabies dispensary to stop the PEP. It is important to note that many persons who have been referred to the anti-rabies dispensary by the veterinary services will never be seen by a medical facility and will then remain unvaccinated.

The aim of the present study is to evaluate the extent of the threat that canine rabies represents in the CAR and to determine the dynamics of its causative agent by using data from the National Reference Laboratory for Rabies and the anti-rabies dispensary for the year 2012.

## Methods

### Study area

The CAR had an estimated population of 4,487,000 inhabitants in 2011 [20]. The country is divided into 16 administrative prefectures. Its capital and most populous city is Bangui with an estimated population of 740,000 inhabitants in 2011 [20]. Bangui is divided into eight urban districts and subdivided into 205 neighborhoods (or quartiers). Two conurbations surround Bangui: the cities of Bimbo (the country's second-largest city) and Bégoua.

### Study samples and data from rabid dogs

Post-mortem samples of brains from dogs with suspicious behaviors were routinely collected by the veterinarians and the livestock technicians of the ANDE through passive surveillance. These samples were provided to the National Reference Laboratory for Rabies at the Institut Pasteur in Bangui for routine rabies diagnosis by direct fluorescent antibody test (FAT) and polymerase chain reaction (PCR). Each sample was accompanied by a form with the following information about the suspicious dog: sex, location where the dog was found dead, captured and/or killed, name and address of its owner when identified, circumstances of the capture and/or reason why it was killed, and results of the rabies diagnostic tests. Anonymized data collected between January 1st and December 31^st^, 2012 were retrospectively reviewed.

### Data collection from rabies exposed humans

Owners of dogs suspected of being rabid and persons exposed to these animals were referred to the anti-rabies dispensary located at the Institut Pasteur in Bangui to receive the post-exposure treatment upon a decision by a veterinarian or a livestock technician of the ANDE based on the type (mainly bites, scratches, and licks) and circumstances of the exposure (for instance, attack by the dog without any reason). This treatment consists of local treatment of the wound through the cleansing and disinfection of the wound (a tetanus shot is also often provided), followed by a rabies vaccination regimen (Verorab, a purified vero cell rabies vaccine made by Sanofi Pasteur, Lyon France) when prescribed. The schedule used was the WHO-approved abbreviated multisite schedule or 2-1-1 regimen [21]. According to this schedule, two doses were given at day 0 (one in the right arm and one dose in the left arm), one dose on day 7 and one dose on day 21. This schedule induces an early antibody response and is considered effective when post-exposure treatment does not include administration of rabies immunoglobulin [21]. Anonymized data on the rabies exposed humans (age, gender, and origin), exposure (number and type of wound, depth and sites) and delay to consult collected between January 1^st^ and December 31^st^, 2012 were retrospectively reviewed. In addition, data on the rabies vaccination status of the dogs involved, when known, was also collected.

### Ethics statement

This was a non-research national public health surveillance activity approved by the Ministry of Public Health, Population and the Fight against AIDS of the CAR. Approval by institutional review board or written informed consent was not required. Data concerning the exposed humans and/or suspicious dog owners were anonymized before analysis.

### Rabies diagnosis

At the National Reference Laboratory for Rabies, direct FAT was routinely done using an anti-RABV nucleocapsid fluorescent conjugate (Bio-Rad, USA). For diagnostic purposes, PCR was also routinely performed using primers that target a portion of the RNA-dependent RNA polymerase-coding region. Extraction of viral RNA from the original fresh brain samples was done using QIAamp Viral RNA Mini Kit (Qiagen) according to the manufacturer’s instructions.

### Molecular analysis of isolated viruses

To investigate the genetic diversity of RABV circulating in the CAR, we amplified and sequenced a portion of the N gene of 606 nucleotides in length from rabies-positive specimens [22]. The gene of the nucleoprotein has been extensively used for molecular phylogenetic studies because of its relatively conserved variation among reservoir-associated variants and geographic lineages [23]. The date of sampling and location (city or neighborhood if the city is Bangui) of the owners of rabid dogs were available for the majority of these sequences. Sequences were then compared with reference sequences from GenBank, and phylogenetic relationships and geographic distribution were determined. Phylogenetic analysis was conducted in MEGA5 [24].

### Statistics

All statistical analysis was performed using STATA version 11.1 (StataCorp, TX). Categorical variables were compared by Chi-square or Fisher exact test according to the headcounts, continuous variables where compared with Student t-test or Kruskal-Wallis test when appropriate (two-sided, significance assigned at P<0.05). To analyze the spatiotemporal association between reported human exposures and detection of rabid dogs, each geographic unit (for Bangui and its suburbs: the eight urban districts and the cities of Bimbo and Bégoua, and for the rest of the country: the prefectures) by epidemiological week (i.e. weeks of the year numbered sequentially from one to 52 –week one corresponding to the first complete week of the year) was considered as a spatiotemporal unit. We performed Spearman rank correlation tests to examine the relationship between the number of reported human exposures (according to the date of exposure) and rabid dogs (according to the date of death) by spatiotemporal unit. As rabies is a communicable disease, the number of case within a same geographic area is likely to be correlated over time. We used a generalized estimating equations (GEE) approach with a Poisson distribution to confirm the significant association between the number of exposed humans (dependent variable) and the number of reported dogs (independent variable) while taking into account the autocorrelation of data over time. GEE, extension of the generalized linear model, is a population-averaged approach that accounts for the correlation between observations by introducing a working correlation matrix and by using robust variance estimators. The model used has been fully described elsewhere [25]. The correlation matrix can be arbitrarily parameterized, and we choose here a first order autoregressive structure to model the correlation of weekly number of cases in each location. This structure is indicated for time series data when two measurements that are right next to each other in time are pretty correlated, but that as measurements get farther and farther apart they are less correlated. Finally, we took into account the overdispersion of the data by adding an overdispersion scaling parameter in the model [26].

## Results

### Description of the rabid dogs population

**Fig 1:**
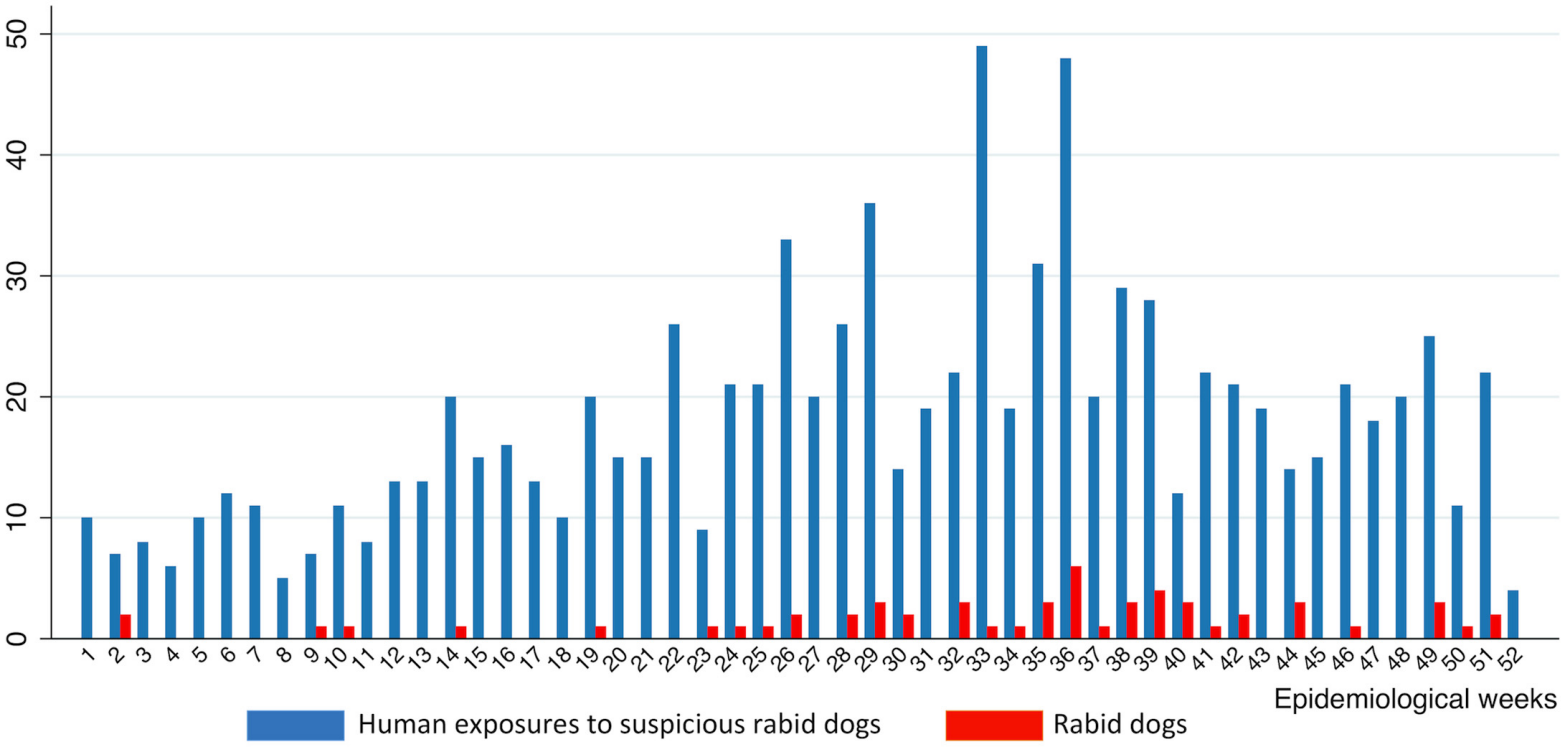
Weekly distribution of the 69 canine rabies cases and the 966 human exposures to suspicious rabid dogs in the CAR in 2012. In dark blue, the weekly numbers of persons exposed to suspicious rabid dogs, and in red, the weekly numbers of rabid dogs.

After only four positive samples by direct FAT in 2011 of seven suspicious samples, the CAR has experienced an important recrudescence of canine rabies in 2012. Of the 83 samples from suspected rabid animals received by the National Reference Laboratory for Rabies, 82 were from dogs and 69 were tested positive with direct FAT (Fig 1 and S1 Table). The remaining sample was from primate and tested negative. Of these 69 samples, 67 were positive with PCR for diagnostic purposes. Characteristics of the 82 suspected rabid dogs that contributed to the samples panel is summarized in the Table 1. Most of the dogs were male (67.3% of the dogs for which the sex was known) with a known owner (68.3%), originated from Bangui (79.3%) and were found aggressive then killed by the owner or by the veterinary services (65.9%).

**Table 1:** Characteristics of the 82 the dogs suspected of being rabid that contributed to the samples panel analyzed at the National Rabies Laboratory during 2012.

Variables	N (%)
**Sex**	
Male	37 (45.1%)
Female	18 (22.0%)
Unknown	27 (32.9%)
**Owner**	
Known	56 (68.3%)
Unknown	26 (31.7%)
**Location**	
Bangui	65 (79.3%)
Prefectures	16 (19.5%)
Unknown	1 (1.2%)
**Outcome**	
Killed	54 (65.9%)
Already dead	26 (31.7%)
Unknown	2 (2.4%)
**Rabies diagnosis**	
Direct IF positive	69 (84.1%)
PCR positive	67 (81.7%)

### Description of the exposed human population

In 2012, a total of 966 persons visited the anti-rabies dispensary for rabid animals exposure mainly after being prescribed to do it by a veterinarian or a livestock technician of the ANDE (Fig 1 and S2 Table). Of these, 631 received the post-exposure vaccines course (regimen 2-1-1). The characteristics of these persons are summarized in Table 2. Briefly, 53.8% were male and 57.7% of the exposed persons were children under 15 years (median age = 13 years with inter-quartile range of 8–27 years) if documented. The main type of exposure was bite by suspicious rabid dogs (91.7%) with multiple (56.0%) but superficial (54.2%) wounds if documented. The vast majority of exposed persons originated from Bangui itself or its suburbs i.e. Bimbo and Bégoua (93.2%). Most of the exposed persons visited the anti-rabies dispensary within a week after exposure (74.8%) but 31 persons came only a month or more after exposure. The time delay to visit the anti-rabies dispensary since exposure was significantly associated with the location of the exposed persons, with a mean delay of 6.2 days for the exposed persons from Bangui, 9.9 days for the persons from Bimbo and Bégoua, and 18.1 days for the persons from the other prefectures (p-value < 0.001). Exposed males were older and more likely to have multiple wounds. Children were significantly more likely to be wounded on the face or the trunk but less on the lower limb, while adults were significantly more likely to be bitten on the lower limb (p-values < 0.001) (Table 2). In addition, persons exposed were asked about the rabies vaccination status of the dogs involved in the exposure. Fifty-eight persons declared to have been exposed to a dog vaccinated but only five of them were able to give details as brand name of the vaccine and/or date of vaccination.

**Table 2:** Characteristics of the 966 persons who have visited the anti-rabies dispensary at the Institut Pasteur in Bangui in 2012 after exposure to suspicious dogs and circumstances of the exposure. For each variable, the results concerned the individuals for whom the information is known.

Total sample		By gender			By age group				
Variables	N (%)	Female	Male	p	<10	10–19	20–39	40+	p
**Gender**									
Male	520 (53.8%)	-	-		-	-	-	-	
Female	446 (46.2%)	-	-		-	-	-	-	
**Age (years)**									
<10	216 (33.8%)	115 (38.6%)	101 (29.5%)	**0.021**	-	-	-	-	
10–19	194 (30.3%)	87 (29.2%)	107 (31.3%)		-	-	-	-	
20–39	148 (23.1%)	55 (18.5%)	93 (27.2%)		-	-	-	-	
40+	82 (12.8%)	41 (13.8%)	41 (12.0%)		-	-	-	-	
**Origin**									
Bangui	810 (84.2%)	433 (84.7%)	377 (83.8%)	0.863	176 (81.9%)	160 (82.5%)	131 (88.5%)	72 (87.8%)	0.451
Bimbo. Bégoua	87 (9.0%)	47 (9.0%)	40 (9.1%)		20 (9.3%)	17 (8.8%)	7 (4.7%)	3 (3.7%)	
Other	65 (6.8%)	37 (6.3%)	28 (7.2%)		19 (8.8%)	17 (8.8%)	10 (6.8%)	7 (8.5%)	
**Type of exposure**									
Nonbite (e.g. scratches and licks)	81 (8.4%)	28 (6.3%)	53 (10.2%)	**0.029**	14 (6.5%)	20 (10.3%)	15 (10.1%)	4 (4.9%)	0.273
Bite	885 (91.7%)	418 (93.7%)	467 (89.8%)		202 (93.5%)	174 (89.7%)	133 (89.9%)	78 (95.1%)	
**Number of wound**									
Unique	291 (44.0%)	141 (44.1%)	150 (43.9%)	0.958	58 (39.7%)	49 (36.6%)	30 (30.9%)	18 (30.5%)	0.435
Multiple	371 (56.0%)	179 (55.9%)	192 (56.1%)		88 (60.3%)	85 (63.4%)	67 (69.1%)	41 (69.5%)	
**Depth**									
Superficial	325 (54.2%)	155 (54.6%)	170 (53.8%)	0.848	55 (42.0%)	56 (49.1%)	35 (45.5%)	19 (35.2%)	0.362
Profound	275 (45.8%)	129 (45.4%)	146 (46.2%)		76 (58.0%)	58 (50.9%)	42 (54.5%)	35 (64.8%)	
**Site of wound** ^a^									
Face	33 (3.6%)	14 (3.3%)	19 (3.9%)	0.621	17 (8.1%)	8 (4.4%)	1 (0.7%)	0	**<0.001**
Upper limb	262 (28.4%)	125 (29.0%)	137 (27.8%)	0.697	81 (38.2%)	60 (33.0%)	46 (33.3%)	17 (21.3%)	0.057
Lower limb	584 (63.3%)	274 (63.6%)	310 (63.0%)	0.859	95 (44.8%)	110 (60.4%)	93 (67.4%)	65 (81.3%)	**<0.001**
Trunk	117 (12.7%)	58 (13.4%)	59 (12.0%)	0.513	43 (20.3%)	23 (12.6%)	10 (7.2%)	3 (3.8%)	**<0.001**
**Delay to consult**									
≤3days	497 (51.6%)	228 (51.2%)	269 (51.9%)	0.955	120 (55.8%)	101 (52.1%)	70 (47.3%)	45 (54.9%)	0.332
4 to 7 days	223 (23.2%)	105 (23.6%)	118 (22.8%)		48 (22.3%)	42 (21.6%)	31 (20.9%)	12 (14.6%)	
>7 days	243 (25.2%)	112 (25.2%)	131 (25.3%)		47 (21.9%)	51 (26.3%)	47 (31.8%)	25 (30.5%)	
**Vaccinal status of the animal**									
Unknown	580 (60.0%)	260 (58.3%)	320 (61.5%)	0.591	167 (77.3%)	144 (74.2%)	109 (73.6%)	53 (64.6%)	0.294
Unvaccinated	328 (34.0%)	158 (35.4%)	170 (32.7)		45 (20.8%)	47 (24.2%)	34 (23.0%)	25 (30.5%)	
Vaccinated	58 (6.0%)	28 (6.3%)	30 (5.8%)		4 (1.9%)	3 (1.5%)	5 (3.4%)	4 (4.9%)	

^a^ The data concerning the site of wound were available for only 922 persons for the face, 923 persons for the (upper and lower) limbs and 924 persons for the trunk. Several sites of wound were possible for a same individual. For this variable, the p-values correspond to the significance level of having a wound versus not having a wound at the concerned site, according to the sex or the age group.

### Spatiotemporal patterns of canine rabies cases and human exposures to rabid dogs

**Fig 2:**
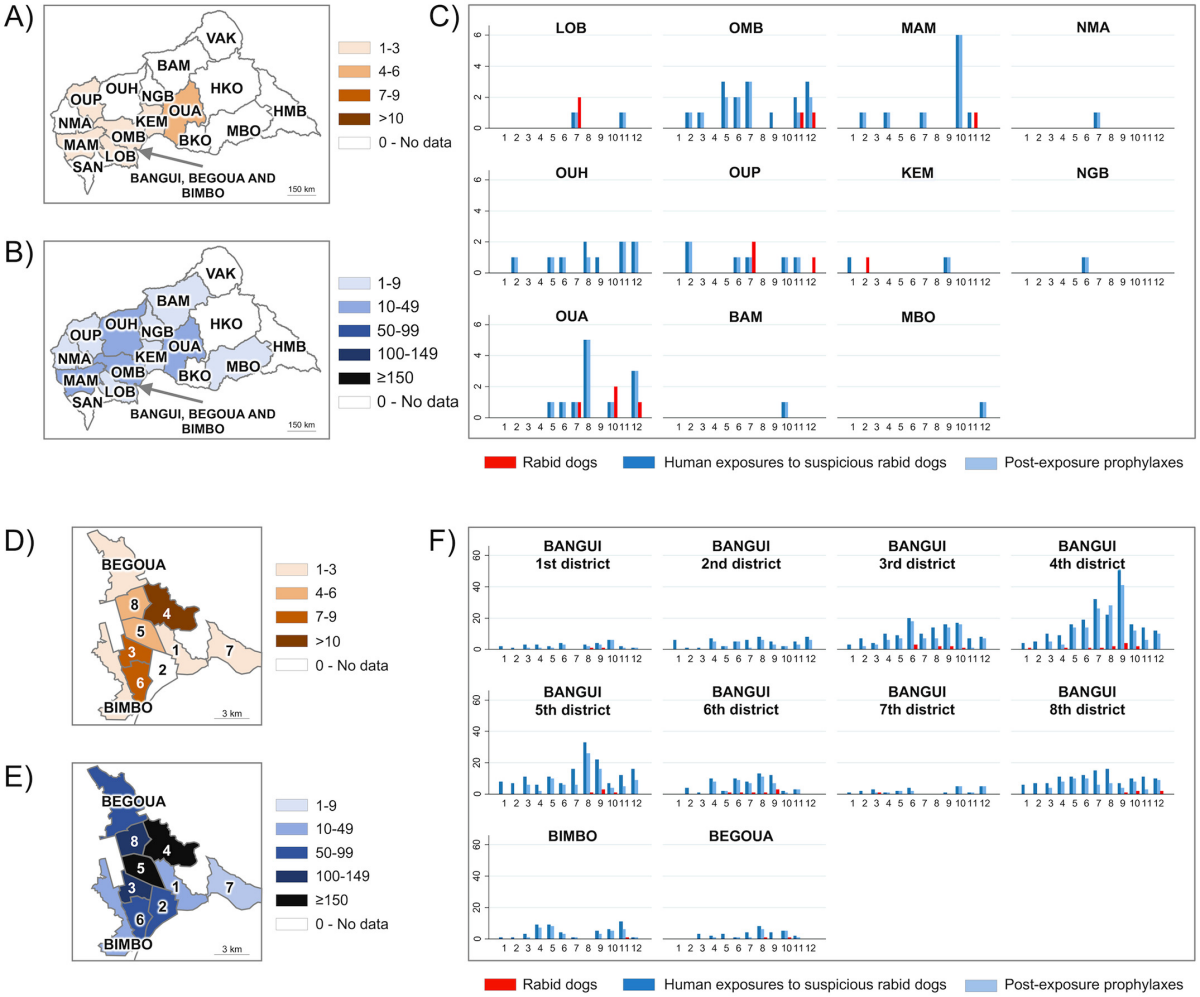
Spatial-temporal distribution of the 69 reported rabid dog cases, the 966 human exposures to suspicious rabid dogs, and the 632 post-exposure prophylaxes in the CAR in 2012. In A), the spatial distribution of reported rabid dogs, in B), the spatial distribution of human exposures to suspicious rabid dogs, and in C), the numbers of reported rabid dogs, persons exposed to suspicious rabid dogs and post-exposure prophylaxes by month in the 16 administrative prefectures of the CAR (BAM: Bamingui Bangoran, BKO: Basse Kotto, HKO: Haute Kotto, HMB: Haut Mbomou, KEM: Kémo, LOB: Lobaye, MAM: Mambéré Kadéï, MBO: Mbomou, NGB: Nana Grébizi, NMA: Nana Mambéré, OMB: Ombella M'Poko, OUA: Ouaka, OUH: Ouham, OUP: Ouham Pendé, SAN: Sangha Mbaéré and VAK: Vakaga). In D), the spatial distribution of reported rabid dogs, in E), the spatial distribution of human exposures to suspicious rabid dogs, and in F), the numbers of reported rabid dogs, persons exposed to suspicious rabid dogs and prophylaxes by month in the eight urban districts of Bangui (1 to 8) and in Bégoua and Bimbo. In red, the rabid dogs, in dark blue, the persons exposed to suspicious rabid dogs, and in light blue, the persons who received a prophylaxis.

The Fig 2 summarizes the data on rabies surveillance in the CAR in 2012. Thirteen dogs were diagnosed as having rabies of 16 samples received from outside of Bangui and its suburbs. They originated from six different prefectures: Lobaye, Ombella M’Poko, Mambéré Kadéï, Ouham Pendé, Kémo and Ouaka (Fig 2A). Most of the persons who were exposed outside of Bangui and its suburbs came from the following prefectures: Ombella M’Poko (17 exposures), Ouaka (12 exposures), and Ouham and Mambéré Kadéï (10 exposures both) (Fig 2B). Most of the canine rabies cases that occurred outside of Bangui and its suburbs were confirmed between July and December (12 cases of 13). Most of the human exposures that occurred outside of Bangui and its suburbs were reported between June and December with 52 exposures of 64 (Fig 2C). In Bangui and its suburbs, most of the canine rabies cases were from the 4^th^ (with 13 rabid dogs), the 3^rd^ and the 6^th^ districts of Bangui (with seven reported rabid dogs both) (Fig 2D). Most of the persons who were exposed in Bangui and its suburbs were from the 4^th^ district (216 exposures), the 5^th^ district (159 exposures), and the 3^rd^ and the 8^th^ districts (129 and 123 exposures, respectively) (Fig 2E). Most of the canine rabies cases that occurred in Bangui and its suburbs were confirmed between June and October (43 cases of 55) while most of the human exposures were reported during the months of June (86 cases), July (95), August (118) and September (138 cases) (Fig 2F). In the most affected districts, the peak happened in June for the 3^rd^ district (20 exposures), in August for the 5^th^ and the 8^th^ districts (33 and 16 exposures respectively), and in September for the 4^th^ district (56 exposures).

**Fig 3:**
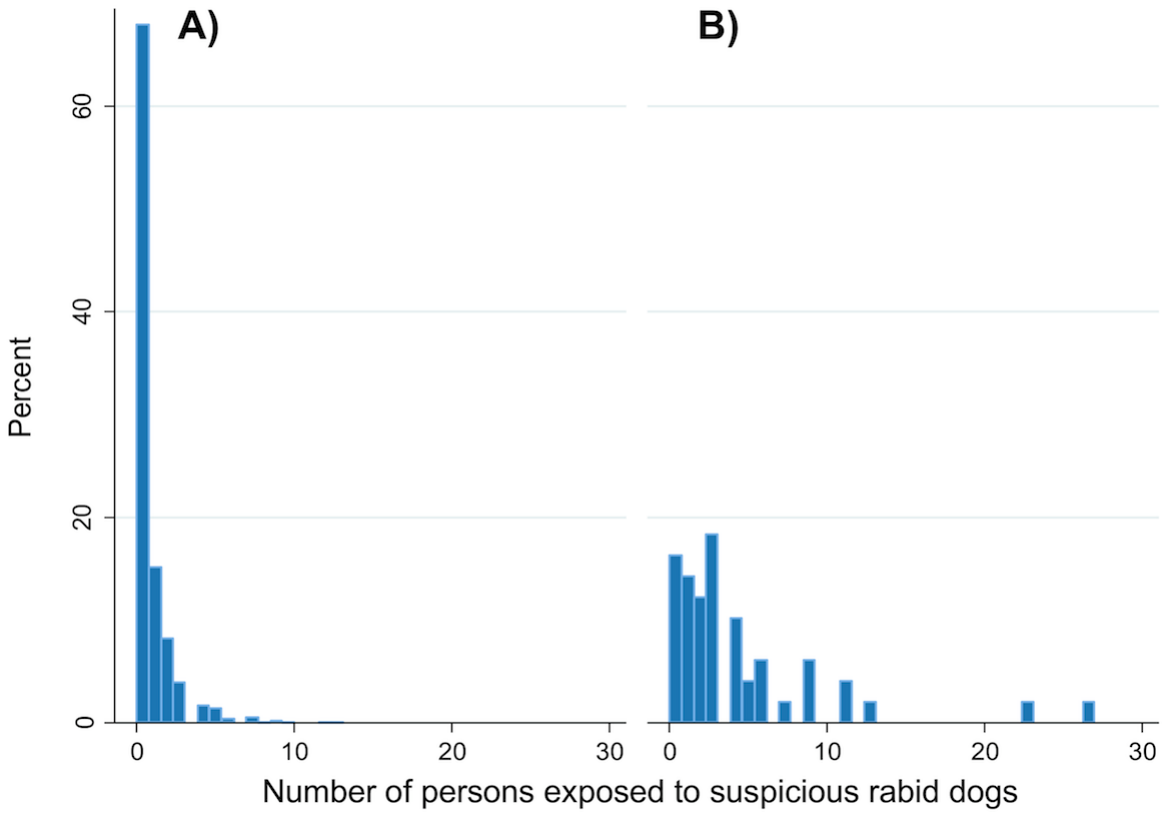
Distribution of the number of persons exposed to suspicious rabid dogs according to the presence of rabid dogs reported the same week in the same area. In A), if no rabid dog was identified in the same area the same week, and in B), if at least one rabid dog was reported in the same area the same week.

These data suggest that the CAR was hit by an epidemic of canine rabies with a peak that happened during the second half of the year 2012 (Figs 1 and 2). We found a mean number of 0.7 (standard deviation 1.4) persons exposed when no rabid dog was reported the same week in the same area, against 4.3 (standard deviation 5.4) persons exposed when at least one rabid dog was reported in the same week in the same area. We found a significant correlation between the number of rabid dogs and the number of persons exposed (Spearman Rho 0.28, P <0.001) by spatiotemporal unit (Fig 3). The correlation was also significant with the number of persons who eventually received PEP (Spearman Rho 0.31, p-values < 0.001). The associations were confirmed when using a model taking into account the temporal autocorrelation (β = 0.80 and p-value < 0.001 for the number of persons at risk; and β = 1.09, p-value < 0.001 for the number of persons having received post-exposure prophylaxis).

### Phylogenetic analysis and spatial distribution of the circulating strains

**Fig 4:**
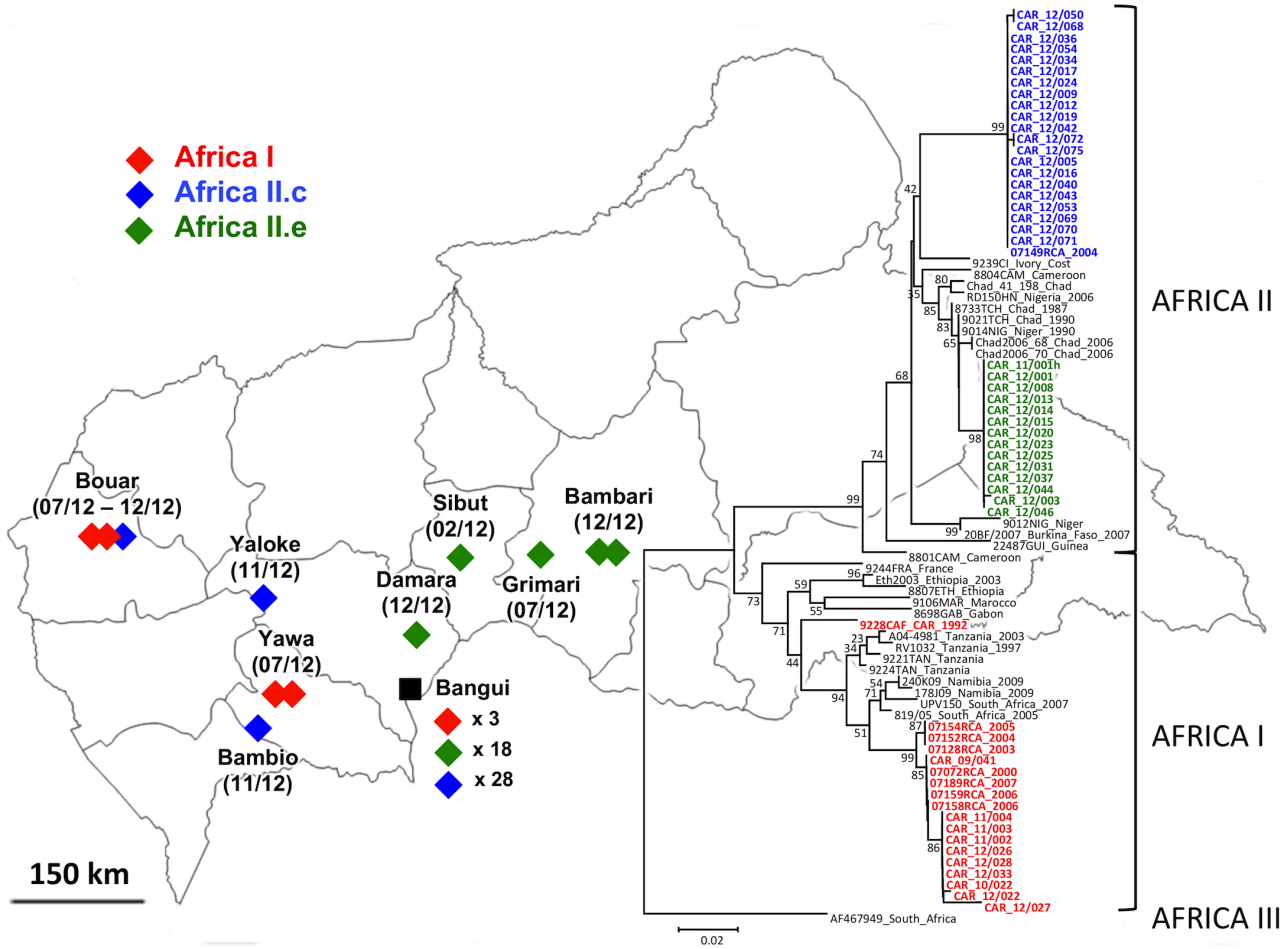
Molecular phylogenetic analysis of representative RABV isolated in the CAR during the year 2012 and their geographical distribution. The neighbor-joining method was used for reconstructing the tree of the N gene sequences of reference RABV strains from Africa I, II and III clades and new sequences from the CAR identified in this study. The percentages of replicate trees in which the isolates clustered together in the bootstrap test (1000 replicates) are shown next to the branches.

From the 67 PCR positive rabid dog samples, 62 sequences of the N gene were obtained [GenBank: KF34677 to KF734738]. These sequences were grouped into three variants based on their similarity. The phylogenetic analysis showed that these variants clustered within two clades: Africa I and II (Fig 4). The Africa I virus isolates described in this study (seven samples) are close to viruses isolated in the CAR in 2000, and in 2003 to 2007 (identity ≥ 99%) indicating that regular circulation of this variant, though a minority in 2012, over the time since 2000. Among the eight main groups of Africa II RABV, the strains isolated in 2012 from the CAR fell within groups c (32 samples) and e (23 samples) [16]. The Africa II group c isolates are close to viruses isolated from Chad, Ivory Coast, Mauritania, Mali Burkina Faso and Senegal (identity around 96–97%). The Africa II group e viruses are close to viruses isolated in Chad, Niger and Nigeria (identity ≥ 98%). In Bangui, all these three variants were found but the Africa I viruses were a small minority with only three samples. Outside of Bangui, these three variants were found having a specific geographic distribution. The Africa I and Africa II isolates were only found in the west of the CAR while the Africa II group e viruses were only found north-east of Bangui (Fig 4).

## Discussion

The main findings of our study highlight that RABV circulated actively in the CAR in 2012 in the absence of any public health intervention to stop its transmission. They also highlight that canine rabies had an important impact on the human health as shown by the hundreds of exposed persons who received PEP. Our phylogenetic analysis has indicated that strains characterized several years before were still circulating together with new strains that were recently introduced as suggested by Nakouné et al [18].

Even if our results concerning the human exposures, rabid dogs and their distribution in the country are expected to be highly under-estimated and biased toward Bangui and its surroundings, they provide an overall picture of the severe situation of the CAR for rabies. These results are in accordance with results from other studies. Bangui has carried an important disease burden. This could partly be because Bangui concentrates an important part of the country population and is where the node of the main trunk roads lies. The most affected areas in Bangui are the most populated districts except the 8^th^ district but its proximity to other very affected districts may explain that this district was also heavily affected. Using an approach similar to Tenzin et al., we have estimated the annual incidence of exposure to rabid dogs in Bangui at 109 per 100,000 population [27]. We found that children were over-represented compared to the adults amongst the potential human exposures to rabies. This has been observed in other African settings [28]. However, without reliable data concerning the population age structure in the area, this is difficult to ascertain if children are at greater risk because the population is likely to be young. In accordance with Kayali et al., most of the rabid dogs found in the CAR were free-roaming and not vaccinated against rabies although they were owned [29]. It is surprising but not uncommon that no confirmed human rabies cases were reported despite the number of rabid dogs reported in 2012, but this may be explained by a likely under-reporting of human rabies cases due to possible overlap of symptoms with those of other infections that could lead to misdiagnosis [30, 31]. It is also possible that human rabies deaths in Bangui have remained unnoticed due to limited ability to investigate relatively rare causes of death. In a recent study in N’Djamena, Chad the annual incidence of human rabies deaths was of 0.7 deaths/100,000 inhabitants [32]. It is also likely that more rabies deaths occurred in rural areas than in urban areas [2]. It is important to note however that several suspicious cases have been reported.

Our results showed that Africa I and Africa II group c isolates seemed to be limited to the west of the country while the Africa II group e viruses were only found in the north-east of Bangui. Together with results from the phylogenetic analyses, these data suggest that Africa I and Africa II group c viruses circulated along the trunk roads between Cameroon and Bangui city while the Africa II group e viruses had followed the north-south axis between Chad and Bangui city. These two axes are the two main roads to reach Bangui from neighboring countries. This is in accordance with the fact that rabies is an example of disease introduction and spread that have resulted from the human-mediated movement of animals even if the influence of these movements on the diffusion of RABV is often difficult to quantify [33, 34]. Several studies have also shown correlation between human-dependent transportation routes and the distribution of virus [35, 36]. The simultaneous circulation of several clades within the same geographical area might indeed indicate a gene flow due to human–mediated transport of dogs. This simultaneous circulation has been observed in other countries–for instance, in China and Thailand [23, 35, 37]. Control of dog movements associated with humans is then essential for rabies control and dog mass vaccination performance.

Our study has several limitations. As previously mentioned, our results are likely to be highly under-estimated and biased toward Bangui and its surroundings. Indeed, our data reflect primarily the situation of Bangui and its near surroundings, but little was known about the rabies situation in other urban or rural areas of the CAR. This is due to a very limited access to any anti-rabies dispensary for people living outside of Bangui and its surroundings. This is also due to the difficulty to ship to Bangui the brain samples collected from suspicious animal in the other urban or rural areas of the CAR. Indeed, the absence of anti-rabies dispensaries outside of Bangui, and the distance, poor road conditions and insecurity prevents any effective surveillance and access to PEP for exposed persons in most parts of the country. In addition, exposures in endemic areas are often ignored or deemed as minimal and it is likely that only a fraction of exposed persons has visited the anti-rabies dispensary even in Bangui and its surroundings like in other African countries [28, 38]. Moreover, the dog at the origin of the exposure was not identified for most of the human exposure cases and the circumstances of the exposures were often not well documented. Another limitation of our study is that bias of awareness and auto-correlation were likely to affect our spatiotemporal correlation analyses. Indeed, a recent work in Africa has demonstrated that the intensity of rabies-control efforts seems to depend on the level of perceived prevalence [19]. Despite these limitations, dog-bite injuries have been shown to provide a valuable and accessible source of data for surveillance in countries where case incidence data are difficult to obtain [11]. Indeed numbers of human rabies deaths and post-exposure treatments correlate closely with numbers of confirmed animal cases even though medical and veterinary authorities operate independently [19].

According to our results, there is an urgent need for implementing an effective national strategy plan to control rabies in the CAR. Reducing dog density through mass euthanasia is ineffective to control rabies [39]. We recommend that this plan should be based on dog mass vaccination. This is the most efficient intervention to move towards the rabies elimination and is more cost-effective than exclusive implementation of human PEP [12, 36]. Although there is evidence that some wild canid populations can support rabies cycles in Africa, most outbreaks in wild canids are triggered by epidemics in domestic dogs rather than the converse [40–42]. In Bangui, only 379 dogs were vaccinated in 2012 (data from the national veterinary services) while the dogs population is likely to be over several tens of thousands of dogs if compared with other capital cities in the region [43]. Effective vaccination campaigns need to reach a sufficient percentage of the population to eliminate disease and prevent future outbreaks, which for rabies is predicted to be around 70% [9, 11]. In addition of achieving sufficient vaccination coverage, regular follow–up campaigns are essential for achieving elimination, especially when achieving high coverage is problematic [36, 44]. An analysis conducted in 2002 and 2006 in N’Djaména, Chad has estimated that to achieve a minimum of 70% of vaccination of the owned animals, the maximum amount that could be charged would be approximately 400–700 CFA francs (1 USD ≈ 483 FCFA) [45]. Today, the charge for dog vaccination at the government-run veterinary clinic in Bangui is 3,500 CFA francs. This is clearly too expensive for average people and the vaccine needs to be provided for free by the government or other funding bodies. Moreover, the four doses of the human vaccine cost 50,000 CFA francs in Bangui but are freely available at the Institut Pasteur in Bangui to every exposed person. It is then likely that implementation of mass dog vaccination campaigns associated with continuation of providing PEP would be more cost-effective than post-exposure vaccination of exposed humans alone in a few years as predicted by Zinsstag et al. [12].

In parallel of implementing dog mass vaccination, the national strategy plan to control rabies should also include some interventions to guarantee a better surveillance and a nationwide improved access to PEP for human exposures to rabies [46]. Indeed, limitations of our study show that there is an urgent need for reinforcement of the national surveillance system, as surveillance is the first step toward an effective control. This could be achieved by decentralizing the data collection capacities i.e. the diagnostic and anti-rabies health centers, in order to cover the entire country, thus taking a better advantage of the extended network of veterinarians and livestock technicians across the country (122 sentinel sites for veterinary surveillance). This should be accompanied by the implementation of an integrated data reporting system. In addition, the surveillance can also be improved by regular communication campaigns to raise public awareness about this deadly disease. A special emphasis would be placed on the necessity to report to the veterinary services all suspicious dogs, on the importance for the dog owners of having his/her dog vaccinated and the urgency of going to the nearest anti-rabies dispensary in case of exposure to suspicious rabid dog.

An early and appropriate access to PEP should also be guaranteed to every exposed person when needed [46]. Currently, there are too many weaknesses in the management of suspected rabies exposures. For instance, there is no follow-up of persons exposed to suspicious rabid dog who did not come to the anti-rabies dispensary but who have been seen by the veterinary services. A closer cooperation between physicians and veterinarians is needed here [32, 47, 48]. It is also important to strengthen the links between the national rabies laboratory and the anti-rabies dispensary or even merge them into the same entity to enable the identification and proper management of potentially exposed persons for all confirmed rabid dogs. A better use of PEP for the exposed persons is necessary in order to optimize the use of limited resources. This could be achieved through a better training on WHO guidelines and staffing of the dispensary. In case of the involved animal is declared healthy, a good coordination should occur to ensure that the exposed persons and the anti-rabies dispensary are timely informed in order to discontinue the PEP. This information should be clearly and appropriately recorded. In order to reduce PEP cost (currently carried by the Institut Pasteur in Bangui), intradermal regimens that require considerably less vaccine than the intramuscular regimens represent a particularly appropriate method when resources are limited [6–8]. We also recommend to extend the administrations of rabies immunoglobulin to all cases of exposure of category III (i.e. single or multiple transdermal bites or scratches, licks on broken skin, contamination of mucous membrane with saliva from licks) as recommended by WHO [6].

Finally, this national strategy plan will necessitate a strong political commitment driven by a proper awareness on the burden of the disease. Several advocacy activities were carried out towards the Ministry of Health as well as the WHO, UNICEF and some NGOs to support the setup and the sustainable operation and supply of one anti-rabies dispensary in each of the seven health regions of the CAR. However, none were successful. As WHO pointed out: “the under-reporting of rabies also prevents mobilization of resources from the international community for the elimination of human dog-mediated rabies” [5]. In the CAR, human rabies is a notifiable disease since April 2009, but this decision has not been appropriately transmitted to the health facilities, and is therefore not applied. Only a few suspected human rabies cases are reported every year. In this situation, this is difficult to attract attention from Public Health stakeholders or policy makers on this problem. However, in the light of these results, we hope that a national strategy plan based on our recommendations will be set up in a near future and run with enough political willingness to effectively control this neglected disease that mostly affects the poor, and is a glaring example of global inequalities in health care.

### Conclusion

Taken together, these data indicate that canine rabies was endemic in the CAR in 2012 and had an important impact on the human health, as shown by the hundreds of exposed persons who have received PEP. The vaccine coverage of domestic dogs against rabies was very low. Implementation of effective public health interventions including mass dog vaccination but also the improvement of the surveillance and the access to PEP is urgently needed to control rabies in the CAR.

## Supporting Information

S1 Table Database on study samples collected from the 82 dogs suspected of being rabid in the CAR in 2012. (TXT)

S2 Table Data on the 966 persons who have visited the anti-rabies dispensary at the Institut Pasteur in Bangui in 2012 after exposure to suspicious dogs and circumstances of the exposure. (TXT)
